# Physical Chemical and Textural Characteristics and Sensory Evaluation of Cookies Formulated with Date Seed Powder

**DOI:** 10.3390/foods11030305

**Published:** 2022-01-24

**Authors:** Zein Najjar, Maitha Alkaabi, Khulood Alketbi, Constantinos Stathopoulos, Meththa Ranasinghe

**Affiliations:** 1 Department of Food Science, College of Agriculture and Veterinary Medicine, United Arab Emirates University, Al Ain P.O. Box 15551, United Arab Emirates; zeinrnajjar@gmail.com (Z.N.); 201401011@uaeu.ac.ae (M.A.); 201401097@uaeu.ac.ae (K.A.); 201990013@uaeu.ac.ae (M.R.); 2 Faculty of Agrobiology, Food and Natural Resources, Czech University of Life Sciences Prague, 165 00 Prague, Czech Republic

**Keywords:** waste utilisation, date seed powder, cookies, sensory analysis

## Abstract

Date seeds are a major waste product that can be utilised as a valuable and nutritional material in the food industry. The aim of the present study was to improve cookies quality in terms of functional and textural value and assess the effect of date seed powder flour substitution on the physical and chemical characteristics of cookies. Three substitution levels (2.5, 5 and 7.5%) of flour by fine date seed powder from six varieties locally named *Khalas*, *Khinaizi*, *Sukkary*, *Shaham*, *Zahidi* and *Fardh* were prepared. Two types of flour were used (white flour and whole wheat) at two different baking temperatures: 180 and 200 °C. The incorporation of date seed had no or slight effect on moisture, ash, fat and protein content of the baked cookies. On the other hand, incorporation significantly affected the lightness and hardness of cookies; the higher level of addition, the darker and crispier the resulting cookies. The sensory analysis indicated that the produced cookies were acceptable in terms of smell, taste, texture and overall acceptability. The results indicate that the most acceptable cookies across all evaluated parameters were produced using whole wheat flour with 7.5% levels of date seed powder using *Khalas* and *Zahidi* varieties. Overall, the analysis indicated that cookies with acceptable physical characteristics and an improved nutritional profile could be produced with partial replacement of the white/whole wheat flour by date seed powder.

## 1. Introduction

The arid and semiarid regions of countries in North Africa and the Middle East are good habitats for the date palm plant [1,2,3,4]. Date palm is of economic and social importance for people in date-producing countries [5]. The pericarp of the date fruit is the edible part, and the pit is a waste product [3]. Date pits, on average, make up 10–15% of the total date fruit’s mass, depending on the variety [6]. Dates of low quality and a hard texture, as well as contaminated ones, are rejected and sometimes used for feed in the animal and poultry industries [7]; up to 800,000 tonnes/year of seeds could be disposed of [2]. Date seeds are composed of 2.10–7.10% moisture, 2.3–6.4% protein, 5–13.2% fat, 0.9–1.8% ash and 72.5–80.2% dietary fibre [8].

Cookies hold an important position as snack foods due to the variety of taste, texture and digestibility [9,10,11,12]; they are also one of the most popular bakery products consumed by almost all levels of society due to their long shelf-life and low cost [13,14]. To improve the nutritive value of cookies, they can be prepared with fortified or composite flour [10,11,12,15,16]. Incorporating date seed powder into baked goods has been tried previously [17,18,19,20] with promising results. Such incorporation would not only improve the nutritional value of the produced cookies, for instance, by enhancing dietary fibre content and antioxidant properties, but also contribute to the utilisation of a waste product of regional significance, date seeds.

In most of the date-processing plants, including date confectionery and date syrup, the seed is considered as the major waste product which amounts to approximately 10% of the fruit mass. Thus, the utilisation of date waste is important for date cultivation and to increase the income in date-processing units. Currently, seeds are used mainly for animal feed for various livestock and sometimes as a soil additive [2,21]. The prevailing global trends aim for a more circular economy. In this context, recently developed food recovery hierarchies in Europe and US highlight the idea that priority should be given to formulating human diets rather than animal feed [22,23]. According to this point of view, there is an increased interest in sustainable and healthier diets along with building up a circular economy. Therefore, the main focus is the valorisation of by-products by incorporating them into food formulations, hence enabling their re-entry into the food chain as new products [24,25].

At the same time, baked food items such as cookies, biscuits, muffins and cakes are very popular among consumers due to their taste and availability. When considering the health aspects, they are usually high in sugar, whereas they are low in antioxidants, minerals and fibre content [26]. Hence, incorporating a by-product, such as date seeds, into the baked foods can be recognised as a perfect solution to improve the food quality as well as to reduce the impact of food processing waste.

Therefore, the purpose of this study was to determine the physicochemical and sensory attributes of date seed powder (DSP)-substituted cookies in order to enable the development and production of better cookies in terms of quality and nutritional value while reducing waste.

## 2. Materials and Methods

### 2.1. Materials

Date seed powder of six fully ripe Emirati date varieties locally known as *Khalas, Khinaizi, Sukkari, Shaham, Zahidi* and *Fardh* were used. The seeds were collected from local farms located in Al Ain, United Arab Emirates. Flour (whole wheat flour—12% protein; white flour—13% protein), sugar, salt, palm oil, food-grade ammonium bicarbonate (NH_4_HCO_3_) and food-grade sodium bicarbonate (NaHCO_3_) were purchased from a local market (Al Ain, United Arab Emirates).

### 2.2. Samples Preparation

#### 2.2.1. Date Seed Powder

The received seeds were cleaned, washed with water, air-dried, ground into powder according to [17] and sieved to obtain fine particles less than 250 µm in diameter. Date seed powder was packed in zip-lock plastic bags and stored at −20 °C until use.

#### 2.2.2. Cookies Preparation

The recipe of cookies described in the American Association of Cereal Chemists (AACC) method 1054 [27] was used with modifications. The original formula was as follows: 80 g white/whole wheat flour, 30 g palm oil (raised to 40 g when using whole wheat flour), 35 g sucrose, 0.8 g NaHCO_3_, 1.0 g salt, 0.4 g NH_4_HCO_3_ and 17.6 g water. The flour was replaced with preparations of date seed powder to a level of 2.5%, 5.0% and 7.5% in the cookie recipe, and cookies with no substitution were produced as control samples. Two types of flour—white (WF) and whole wheat flour (WW)—and two baking temperatures of 180 °C and 200 °C (for 10 and 8 min, respectively) were used, making twelve different combinations for each variety of date seeds. The dough was kneaded and sheeted to a uniform thickness using a pasta machine set at 3 mm. Then, the dough was cut with a stainless-steel cutter into circular shapes of a diameter of 4.5 cm. The cookies were baked in a wall oven (Miele & Cie. KG, Bielefeld, Germany) and were packed in sealed plastic bags and stored at −20 °C until further analysis.

### 2.3. Proximate Composition

Moisture percentage was determined by the oven dry method (Association of Official Analytical Chemists (AOAC) method 934.01) [28] using an oven (Carbolite Gero Limited, Sheffield, UK) at 85 °C for 4 h and ash by direct analysis (AOAC method 940.26) using a furnace (CWF 1100, Carbolite Gero Limited, Derbyshire, UK). Protein was determined by Kjeldahl nitrogen (AOAC method 920.152) [29] using Kjeldahl apparatus (AutoKjeldal unit K-370, BUCHI, Flawil, Switzerland), and protein was calculated using the general factor (6.25). The percentage of crude fat was determined by the Soxtec automated extraction method (AOAC, 2003.05) [28] using the Soxtec Auto fat extraction system (FOSS Analytical, Hillerød, Denmark). All assessments were conducted in triplicate. Carbohydrate content was calculated by difference: 100 − (moisture + ash + protein + fat).

### 2.4. Physical Analysis

#### 2.4.1. Colour Analysis

The colour of the date seed powder of six varieties and cookie samples was determined using a Hunter Colourimeter fitted with an optical sensor (HunterLab ColourFlex EZ spectrophotometer. Hunter Associates Laboratory, Inc., Reston, VA, USA) on the basis of the L*, a*, b* colour system. L* is the lightness component that measures black (0) to white (100), a* parameter goes from green to red, and b* parameter from blue to yellow. Throughout this manuscript, the colour analysis was performed only on the basis of lightness (L*). Six cookies were evaluated for each formulation.

#### 2.4.2. Texture Analysis

Measuring hardness was determined using Texture Analyzer (BROOKFIELD CT3 Texture Analyzer, Brookfield Engineering Labs, Inc., Middleborough, MA, USA) equipped with a 3-Point Bending Rig in a compression mode with a sharp blade-cutting probe (TA7). Pre-test and test speeds were 1 and 0.5 mm/s, respectively, at a trigger load of 3.0 g. Hardness (maximum peak force) was measured with at least 3 cookies for each sample. The peak force, when the cookie began to break, was reported in Newton (N). 

#### 2.4.3. Sensory Evaluation

Cookies were subjected to a sensory evaluation using 30 untrained panellists recruited from within the university community. Sensory properties (taste, aroma, colour, crispiness and overall acceptability) were evaluated. The ratings were on a 9-point hedonic scale ranging from 9 (like extremely) to 1 (dislike extremely).

### 2.5. Statistical Analysis

The data were shown as mean ± standard deviation (SD). Data were assessed by Tukey test using (Minitab 19, USA) statistical software package at *p* < 0.05 to determine the level of significance.

## 3. Results and Discussion

### 3.1. Proximate Analysis

#### 3.1.1. Date Seed Powder Chemical Composition

The chemical composition for DSP varieties used in making cookies is shown in Table 1. Regarding moisture, *Sukkari* seed powder was the least moist, followed by *Zahidi Fardh, Khinaizi, Shaham* and *Khalas* seeds. Differences in cultivars, as well as cultivation under divergent climatic conditions, make it possible to have discrete moisture levels. Previous studies on date seed flour also demonstrated different moisture contents in different varieties [30,31]. For ash content, *Sukkari* seed powder had the lowest, while seeds of the *Fardh* variety had the highest. In terms of crude fat, *Zahidi* seeds had a high amount of fat, opposite to the *Khinaizi* seed variety. No significant difference was observed in protein content between the varieties used. The lowest carbohydrate content was in *Khalas* powder; it was significantly different from *Sukkari, Shaham* and *Zahidi* varieties. In previous findings for *Khalas*, *Sukkari* and *Fardh*, the results are quite different from this study [30,31,32]. These differences of proximate compositions, moisture and ash may be due to the climatic conditions or the variability of the cultivars used, as postulated also by [33]. Data about the chemical compositions of the remaining date seeds varieties—*Khinaizi*, *Shaham* and *Zahidi*—could not be found.

#### 3.1.2. Proximate Composition of Cookies

The percentage of moisture, ash content and crude fat content of different cookie samples are shown in Table 2, Table 3 and Table 4, respectively.

In Table 2, when considering the differences between white flour and whole wheat flour cookies: at 180 °C, the WF cookies consistently had higher moisture content. At 200 °C, the same trend was observed with WF cookies having higher moisture content than the WW composite cookies. The control samples showed the same pattern. We believe that the higher proportion of gluten in WF cookies leads to this effect. The only exception in these observations was the *Khalas* cookies at 2.5% addition, where the WW cookies had higher moisture than the WF ones. The reasons for this difference are not clear.

When considering the differences between baking temperatures (for the same flour type) overall, as expected, the cookies baked at 200 °C had lower moisture content than those baked at 180 °C. This observation was true for the controls as well as the composite cookies. The exceptions were the *Shaham* cookies that exhibited no significant differences between the two baking temperatures.

When considering the differences among varieties, for the 180 °C WF cookies, at 2.5 and 5% addition, *Shaham* had the highest values, while at 7.5%, the highest was *Khalas*. For the 180 °C WW cookies, at all levels of addition, the Khalas variety cookies exhibited the highest values. For WF cookies baked at 200 °C, there was no clear pattern emerging on the basis of date seed variety. For WW cookies baked at 200 °C, *Khalas* showed the highest moisture values at all addition levels.

In previous studies, improved water holding capacities resulted in bread formulated with defatted date seed powder [20] and extracted polysaccharides of date seeds [34]. Therefore, the results in this study could be attributed to the improved water holding capacity [35] due to the high amount of fibre content in date seeds [17,18,19]. When it comes to preservation, convenience in packaging, storage and transport, the moisture content is an important quality factor. Differences in moisture contents among date seed varieties could also be responsible for the discrete levels of moisture in composite cookies.

Ash content differences between cookies are listed in Table 3. Regarding the effect of flour type, in the control samples, WF cookies had lower ash content than WW ones. In composite cookies, however, there was no clear trend observed. With regards to the effect of cooking temperature (for the same flour type), there were no significant differences in the control samples (no DSP addition), while no trend was apparent for composite cookies. Assessing the results by DSP variety, for WF at 180 °C, *Zahidi* cookies had the highest values at all levels of addition. For WW cookies at 180 °C, again, the *Zahidi* variety had the highest values at the higher levels of addition (5% and 7.5%). For WF cookies prepared at 200 °C, the *Zahidi* variety had significantly higher values than the rest, although no significant differences were observed between levels of addition. For WW cookies baked at 200 °C, *Fardh* cookies had the highest values at all levels of addition. Overall, the results regarding the ash content are somewhat surprising as they did not correspond to the ash content of the DSP.

There was no significant improvement in ash content of some of the composite cookies compared to control samples, although a considerable amount of ash was observed in date seed powder, especially *Fardh*. This may be due to the addition of lower amounts of date seed powder in composite cookies, leading to no significant difference in ash content between the control and composite samples. High ash content is an indication of high mineral content. Date seeds are found to be rich in minerals [3,36], and improved mineral content was observed in composite bakery products in a previous study [37]. Therefore, the addition of higher amounts of date seed powder may increase the ash content and hence mineral content in composite cookies.

The fat content results in Table 4, when examined with regards to the flour type used, indicate that for both controls and composite cookies, WF cookies had lower fat content than WW cookies at both temperatures. The addition of DSP did not result in significant differences between controls and composite cookies. When looking at the effect of baking temperature (for the same flour type), no significant differences were observed for both controls and composite cookies. Regarding the effect of DSP variety, no significant differences were observed at any flour-temperature combination. 

The different stages of cookie preparation change the physical and chemical properties of the various flour constituents, including fat, present in the cookie dough formula [38]. Moreover, the fat content in different date seed varieties would result in different levels of fat in the final product. Fat provides a number of benefits to improve the physical and textural quality of cookies [38].

The incorporation of date pits in the form of powder at different levels into cookies resulted in similar fat content, and this can be attributed to the high amount of fat in the oil and flour used (accounting for about 96% of the total fat content of cookies) that masks the fat content coming from date seed powder. The same explains the insignificant difference in the protein content of the cookies, where the protein content came mainly from the high amount of protein in whole wheat and white wheat flour. 

However, using even higher date seed powder addition levels, as shown by Platat et al. [17], may make a significant difference, although the consumer acceptability of such formulations would need to be confirmed. The chemical composition of date seed pits is known to vary depending on the variety and can be attributed to the use of fertilisers, harvest time and post-harvest treatments [30]. Thus, it is quite expected to notice a difference in results among the varieties.

In terms of protein content, the incorporation of date seed powder of any variety in making cookies did not affect the protein content when compared to cookies with zero incorporation, which agrees with Platat et al. [17]. The date seed powders used contained similar levels of protein to the wheat flours used, and, considering the levels of addition, this resulted in non-significant differences in the protein content of the composite cookies. 

### 3.2. Physical Properties of Cookies

#### 3.2.1. Colour Analysis

The colour measurements of date seed powder varieties and the cookies substituted with various date seed powder are listed in Table 5. The lightness of the six varieties used was significantly different from each other; *Sukkari* date seed powder was the lightest in colour, and *Khalas* was the darkest; however, the differences in the level of lightness between the date seed powder did not affect the final colour of the cookies. White flour cookies with no addition of date seed powder were obviously lighter than cookies of whole wheat flour, and the lightness of the composite cookies displayed a decreasing trend with the increasing level of addition; the higher level of addition, the darker the cookie. These results are in accordance with Gómez and Martinez [16], Aksoylu et al. [39] and Ashoush and Gadallah [15], who reported the colour alterations in biscuits due to incorporation of by-products such as seeds. Therefore, the colour of cookies was significantly affected by the DSP addition; the composite cookies were darker, and the dark colour is caused by the natural dark pigmentation of date seeds regardless of the variety.

#### 3.2.2. Texture Analysis

Cookies’ hardness assessments are listed in Table 6. The texture of all cookies made with *Shaham* date seed powder was not affected by the addition level and was almost similar to the texture of *Zahidi* cookies, which were softer when prepared with white flour at 200 °C at an addition level of 7.5%. The effect of the addition level of either *Fardh* or *Sukkari* seed powder on the texture of cookies was different; the more powder added, the softer the cookies. Moreover, the case was the same in *Khalas* cookies, except for whole wheat flour *Khalas* cookies baked at 180 °C, where softer cookies were obtained. While in whole wheat flour *Khinaizi* cookies at any baking temperature, the softness was not affected, differing from white flour *Khinaizi* cookies, where a decrease in hardness with the addition level was recognisable.

With the addition of date seed flour, cookies become softer when the control is white flour, except for *Shahm* and *Zahidi*. In the case of whole wheat flour, a significant difference was noticeable in *Khalas* and *Sukkari* at higher addition levels and the 7.5% addition level in *Fardh* at 200 °C, whereas at 180 °C, it was only in *Fardh* and 7.5% addition level in *Sukkari*.

The most pronounced decrease in the hardness of the obtained composite cookies was observed for the 7.5% addition level of white flour *Sukkari* cookie samples at 200 °C, which showed a prominent significant difference from the control. However, it was not significantly different from *Khinaizi* and *Fardh* at the same treatment conditions. In comparison among varieties, the increase in the softness was quite underlined in *Fardh* cookies in all the treatment conditions at 7.5% inclusion level except white flour *Fardh* cookies at 200 °C. In that treatment condition, all the inclusion levels of 2.5%, 5% and 7.5% were also significantly different from the control, although it did not show significant differences between the addition levels. 

Composite cookies were softer in accordance with similar studies on seed incorporation, such as amaranth flour composite cookies [10,40]. According to Chauhan et al. [10], the decrease in hardness was due to the replacement of wheat flour with the seed flour, which results in a gluten content reduction in the cookie dough which, in turn, contributed to the substantial decrease in hardness. This phenomenon is applicable to the observations in our study. Moreover, several studies on bakery items, such as bread and biscuits, demonstrated that hardness is mainly due to the interactions between gluten and fibre, where dietary fibre leads to higher water absorption and interferes with gluten development time [41,42]. Bouaziz et al. [34] tried incorporating the extracted dietary fibre from date seeds, which resulted in a decrease in bread hardness. Since date seeds are rich in dietary fibre [17,18,19,34,43], this can be a reason for the reduction in hardness with increasing levels of date seeds.

#### 3.2.3. Sensory Evaluation

**Figure 1:**
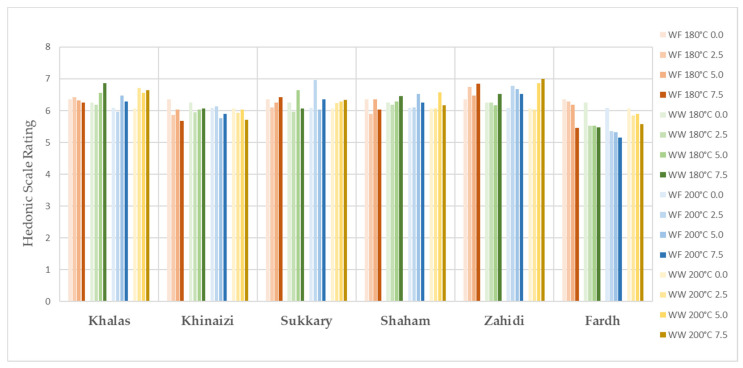
Smell evaluation for date seed composite cookies.

**Figure 2:**
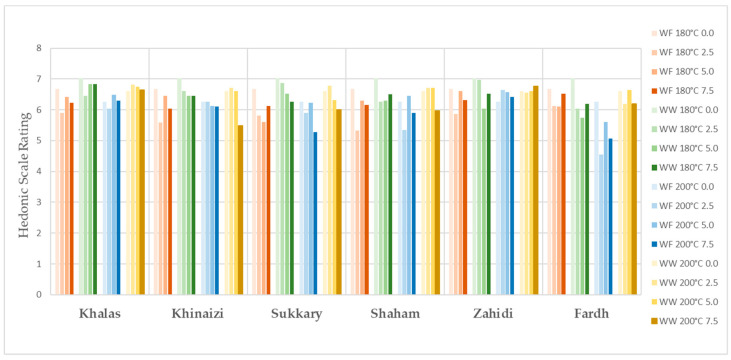
Colour evaluation for date seed composite cookies.

**Figure 3:**
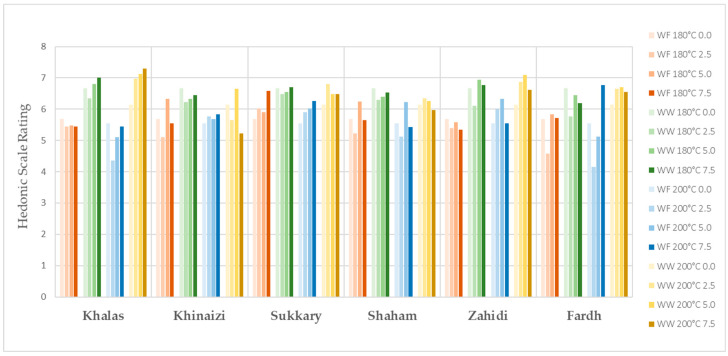
Texture evaluation for date seed composite cookies.

**Figure 4:**
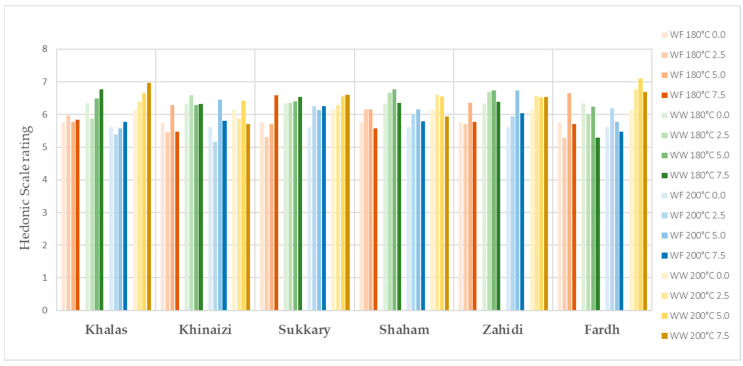
Taste evaluation for date seed composite cookies.

**Figure 5:**
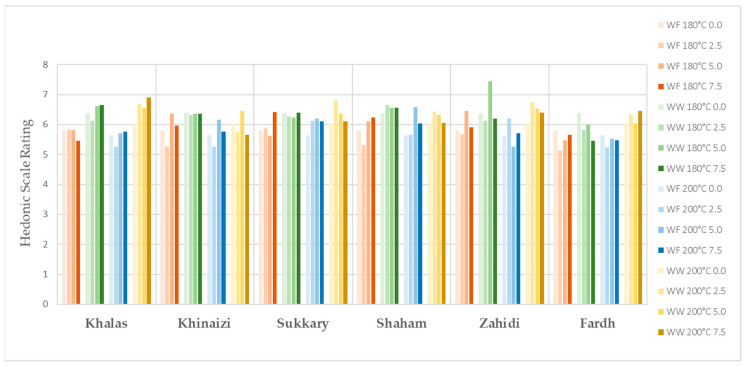
Overall acceptability of date seed composite cookies.

The evaluation of sensory properties of cookies is illustrated in Figure 1, Figure 2, Figure 3, Figure 4 and Figure 5, colour, smell, texture, taste and overall acceptability for all cookies made with different varieties of date seed powder were acceptable and were rated between 5 and 7 on a hedonic scale. 

The consumers’ evaluation in terms of colour, texture and taste showed that the produced cookies were highly acceptable. A darker cookie colour correlates with other physical, chemical and sensorial indicators of product quality. Colour is considered a fundamental physical property of foods and agricultural products, and it affects the assessment of external quality in both the food industry and food engineering research [44,45]. When analysing the results for colour, we cannot say the panellists are only interested in the light colour products, as some scores are higher for the darker ones, such as in the varieties *Khalas* and *Zahidi*. It is noteworthy that in some instances, date seed composite cookies with high substitutions are the ones which are more preferred compared to zero or low-level samples. When the texture is considered, consumer acceptability is high in the samples with high date seed flour substitutions. This may be due to the softness of biscuits. According to the results of hardness, date seed composite cookies have lower hardness compared to the control samples. Hardness is a textural property that plays a major role in the evaluation of baked goods, as it is associated with the human perception of freshness—the lower this parameter is, the more desirable the product [46]. However, the overall acceptability of the date seed composite cookies is significant depending on the variety and the heat treatment.

## 4. Conclusions

This study concluded that the by-products or the waste of date-processing units, such as date seeds, can be used to enhance the quality of cookies in terms of their physical, chemical and textural properties. There were no significant differences in the protein content of cookies with date seed flour incorporated since the composite cookies had a protein content similar to the control samples. For cookies baked at 180 °C, whole wheat flour formulations had lower moisture content than those made with white flour. A similar trend was observed at 200 °C, with whole wheat flour formulations having lower moisture content than white flour for each date variety. Different varieties with different addition levels showed a variation in moisture levels, but at all addition levels, *Khalas’* moisture was the highest. It appears that the moisture content level is primarily affected by the baking temperature and the type of flour, while varietal differences seem to be less pronounced. When considering the ash content, *Zahidi* composite cookies at the 7.5% addition level in both flour types had the highest values. The colour of cookies was significantly affected by the incorporation of date seed flour, making them darker. This may be the reason for the somewhat reduced score for the colour attribute in sensory, even though some addition levels show higher consumer acceptance. However, the sensory analysis results showed that the overall acceptance of the composite cookies was higher than the others with whole wheat flour at both temperature levels. Therefore, based on the sensory analysis, Khalas and Zahidi composite cookies at 7.5% addition level with whole wheat flour can be recommended to obtain the most preferred final product. As expected, an increased softness was observed in date seed composite cookies, except for the *Shaham* and *Zahidi* varieties (where the increment was not significant), leading the path to formulate high-quality cookies in terms of texture.

## Figures and Tables

**Table 1:** Chemical composition of date seed powder ^1^.

Variety	Moisture	Ash	Crude Fat	Crude Protein	Carbohydrate
*Khalas*	5.08 ± 0.10 ^a^	3.31 ± 0.26 ^bc^	9.81 ± 0.37 ^ab^	8.13 ± 2.45 ^a^	73.68 ± 2.20 ^c^
*Khinaizi*	4.06 ± 0.32 ^ab^	3.52 ± 0.26 ^b^	8.26 ± 38.47 ^c^	6.84 ± 0.86 ^a^	77.31 ± 38.44 ^abc^
*Sukkari*	1.24 ± 0.18 ^d^	2.04 ± 0.16 ^d^	9.94 ± 2.98 ^ab^	8.30 ± 1.11 ^a^	78.48 ± 3.61 ^ab^
*Shaham*	4.08 ± 0.58 ^ab^	3.37 ± 0.12 ^bc^	7.70 ± 0.12 ^bc^	6.14 ± 0.38 ^a^	78.70 ± 0.79 ^ab^
*Zahidi*	2.53 ± 0.87 ^c^	2.65 ± 0.04 ^cd^	12.68 ± 0.08 ^a^	6.92 ± 0.46 ^a^	75.22 ± 1.26 ^ab^
*Fardh*	3.72 ± 0.21 ^bc^	6.90 ± 0.63 ^a^	8.03 ± 0.16 ^bc^	7.29 ± 1.12 ^a^	74.06 ± 1.16 ^c^

^1^ Means ± SD are presented. Data expressed as g/100 g on a fresh weight basis, and different lowercase superscript letters in a column denote significant differences, *p* < 0.05.

**Table 2:** Moisture content of cookies ^1^.

Baking Temp, Flour Type, Addition Level		*Khalas*	*Khinaizi*	*Sukkari*	*Shaham*	*Zahidi*	*Fardh*
180 °C White Flour	0	3.25 ± 0.19 ^bc^	3.25 ± 0.19 ^a^	3.25 ± 0.19 ^bc^	3.25 ± 0.19 ^c^	3.25 ± 0.19 ^b^	3.25 ± 0.19 ^a^
	2.5	3.14 ± 0.44 ^cB^	3.03 ± 0.07 ^aB^	4.01 ± 0.27 ^aAB^	4.41 ± 0.13 ^aA^	4.00 ± 0.10 ^aAB^	3.80 ± 0.46 ^aAB^
	5.0	3.85 ± 0.08 ^abA^	3.31 ± 0.28 ^aAB^	3.4 ± 0.36 ^abAB^	3.89 ± 0.24 ^bA^	2.78 ± 0.14 ^cB^	3.55 ± 0.28 ^aA^
	7.5	3.95 ± 0.14 ^aA^	2.90 ± 0.06 ^aBC^	2.66 ± 0.06 ^cC^	3.38 ± 0.06 ^cAB^	2.76 ± 0.18 ^cBC^	3.18 ± 0.47 ^aBC^
180 °C Whole Wheat Flour	0	2.70 ± 0.09 ^b^	2.70 ± 0.09 ^a^	2.70 ± 0.09 ^a^	2.70 ± 0.09 ^a^	2.70 ± 0.09 ^a^	2.72 ± 0.09 ^a^
	2.5	3.42 ± 0.05 ^aA^	2.34 ± 0.13 ^aB^	2.07 ± 0.13 ^bBC^	1.69 ± 0.24 ^bC^	2.09 ± 0.23 ^bBC^	1.07 ± 0.22 ^bD^
	5.0	3.09 ± 0.17 ^abA^	2.20 ± 0.09 ^aB^	1.96 ± 0.13 ^bBC^	2.06 ± 0.05 ^bBC^	1.82 ± 0.09 ^bC^	0.33 ± 0.09 ^bcD^
	7.5	2.98 ± 0.31 ^bA^	1.14 ± 0.46 ^bBC^	1.19 ± 0.08 ^cBC^	1.19 ± 0.13 ^cBC^	1.76 ± 0.19 ^bB^	0.50 ± 0.33 ^cC^
200 °C White Flour	0	2.87 ± 0.11 ^bc^	2.87 ± 0.11 ^a^	2.87 ± 0.11 ^b^	2.87 ± 0.11 ^b^	2.87 ± 0.11 ^a^	2.87 ± 0.11 ^b^
	2.5	3.12 ± 0.08 ^abB^	2.72 ± 0.16 ^aBC^	3.19 ± 0.05 ^aB^	3.89 ± 0.33 ^aA^	2.53 ± 0.03 ^bC^	4.07 ± 0.32 ^aA^
	5.0	2.81 ± 0.04 ^cB^	2.91 ± 0.02 ^aB^	2.67 ± 0.05 ^cB^	3.55 ± 0.24 ^aA^	2.05 ± 0.26 ^cC^	2.45 ± 0.21 ^bcBC^
	7.5	3.30 ± 0.16 ^aA^	2.88 ± 0.07 ^aB^	1.69 ± 0.06 ^dD^	2.89 ± 0.13 ^bB^	2.02 ± 0.04 ^cCD^	2.28 ± 0.22 ^cC^
200 °C Whole Wheat Flour	0	2.06 ± 0.02 ^ab^	2.06 ± 0.02 ^a^	2.06 ± 0.02 ^a^	2.06 ± 0.02 ^a^	2.06 ± 0.02 ^a^	2.06 ± 0.02 ^a^
	2.5	2.52 ± 0.15 ^aA^	2.24 ± 0.28 ^aAB^	1.21 ± 0.04 ^bC^	1.68 ± 0.11 ^bBC^	1.35 ± 0.13 ^bC^	0.62 ± 0.35 ^bD^
	5.0	2.31 ± 0.06 ^abA^	2.20 ± 0.03 ^aA^	1.17 ± 0.05 ^bC^	2.19 ± 0.01 ^aA^	1.40 ± 0.19 ^bB^	0.41 ± 0.00 ^bD^
	7.5	1.95 ± 0.32b ^bA^	1.44 ± 0.38 ^bAB^	0.84 ± 0.17 ^cBC^	1.19 ± 0.27 ^cAB^	1.42 ± 0.23 ^bAB^	0.26 ± 0.38 ^bC^

^1^ Means ± SD are presented. Data are expressed as g/100 g on a fresh weight basis; different lowercase superscript letters in a column and different uppercase superscript letters in a row denote significant differences, *p* < 0.05.

**Table 3:** Ash content of cookies ^1^.

Baking Temp, Flour Type, Addition Level		*Khalas*	*Khinaizi*	*Sukkari*	*Shaham*	*Zahidi*	*Fardh*
180 °C White Flour	0	1.50 ± 0.07 ^a^	1.50 ± 0.07 ^a^	1.50 ± 0.07 ^a^	1.50 ± 0.07 ^a^	1.50 ± 0.07 ^a^	1.50 ± 0.07 ^a^
	2.5	1.68 ± 0.18 ^aA^	1.42 ± 0.07 ^aA^	1.32 ± 0.11 ^aA^	1.87 ± 0.44 ^aA^	1.96 ± 0.04 ^aA^	1.08 ± 0.81 ^aA^
	5.0	1.37 ± 0.43 ^aB^	1.40 ± 0.05 ^aB^	1.42 ± 0.03 ^aAB^	1.54 ± 0.02 ^aAB^	1.95 ± 0.04 ^aA^	1.33 ± 0.23 ^aB^
	7.5	1.46 ± 0.05 ^aBC^	1.53 ± 0.05 ^aB^	1.36 ± 0.04 ^aB^	1.56 ± 0.05 ^aC^	2.07 ± 0.06 ^bA^	0.93 ± 0.01 ^aD^
180 °C Whole Wheat Flour	0	1.63 ± 0.04 ^a^	1.63 ± 0.04 ^a^	1.63 ± 0.04 ^a^	1.63 ± 0.04 ^a^	1.63 ± 0.04 ^a^	1.63 ± 0.04 ^a^
	2.5	1.65 ± 0.04 ^bA^	1.61 ± 0.02 ^aA^	1.65 ± 0.05 ^aA^	1.74 ± 0.12 ^aA^	1.63 ± 0.1 ^aA^	1.62 ± 0.08 ^aA^
	5.0	1.66 ± 0.04 ^bB^	1.66 ± 0.01 ^aB^	1.63 ± 0.06 ^aB^	1.55 ± 0.03 ^aB^	2.08 ± 0.23 ^bA^	1.59 ± 0.09 ^aB^
	7.5	1.81 ± 0.04 ^bB^	1.66 ± 0.03 ^aBCD^	1.53 ± 0.04 ^aCD^	1.60 ± 0.08 ^aD^	2.08 ± 0.12 ^bA^	1.76 ± 0.06 ^aBC^
200 °C White Flour	0	1.32 ± 0.04 ^a^	1.32 ± 0.04 ^a^	1.32 ± 0.04 ^a^	1.32 ± 0.04 ^a^	1.32 ± 0.04 ^a^	1.32 ± 0.04 ^a^
	2.5	1.50 ± 0.12 ^aB^	0.79 ± 0.17 ^bC^	1.22 ± 0.07 ^aB^	1.31 ± 0.26 ^aB^	1.96 ± 0.04 ^abA^	1.55 ± 0.03 ^aB^
	5.0	1.53 ± 0.02 ^aB^	0.84 ± 0.12 ^bD^	1.32 ± 0.1 ^aBC^	1.42 0.04 ^aC^	1.8 ± 0.04 ^bA^	1.49 ± 0.05 ^bBC^
	7.5	1.44 ± 0.14 ^aB^	0.88 ± 0.07 ^bC^	1.33 ± 0.02 ^aB^	1.50 ± 0.11 ^aB^	2.08 ± 0.18 ^cA^	0.79 ± 0.03 ^cC^
200 °C Whole Wheat Flour	0	1.60 ± 0.01 ^a^	1.60 ± 0.01 ^a^	1.60 ± 0.01 ^a^	1.60 ± 0.01 ^a^	1.60 ± 0.01 ^a^	1.60 ± 0.01 ^a^
	2.5	1.53 ± 0.09 ^aAB^	1.12 ± 0.12 ^abC^	1.55 ± 0.04 ^aABC^	1.44 ± 0.03 ^aAB^	1.26 ± 0.26 ^aBC^	1.67 ± 0.08 ^abA^
	5.0	1.59 ± 0.01 ^aAB^	0.99 ± 0.16 ^abC^	1.58 ± 0.08 ^aBC^	1.25 ± 0.17 ^abAB^	1.48 ± 0.05 ^aAB^	1.76 ± 0.18 ^abA^
	7.5	1.67 ± 0.08 ^aA^	0.55 ± 0.42 ^bB^	1.54 ± 0.02 ^aA^	1.55 ± 0.02 ^bA^	1.5 ± 0.03 ^aA^	1.88 ± 0.07 ^bA^

^1^ Means ± SD are presented. Data expressed as g/100 g on a fresh weight basis; different lowercase superscript letters in a column and different uppercase superscript letters in a row denote significant differences, *p* < 0.05.

**Table 4:** Crude fat content of cookies ^1^.

Baking Temp Flour Type and Addition Level		*Khalas*	*Khinaizi*	*Sukkari*	*Shaham*	*Zahidi*	*Fardh*
180 °C White Flour	0	19.34 ± 0.97 ^a^	19.34 ± 0.97 ^a^	19.34 ± 0.97 ^a^	19.34 ± 0.97 ^a^	19.34 ± 0.97 ^a^	19.34 ± 0.97 ^a^
	2.5	19.15 ± 0.49 ^aA^	19.34 ± 1.95 ^aA^	20.71 ± 0.68 ^aA^	19.37 ± 0.15 ^aA^	20.07 ± 0.48 ^aA^	19.65 ± 0.28 ^aA^
	5.0	19.44 ± 0.60 ^aA^	20.60 ± 0.10 ^aA^	19.67 ± 2.60 ^aA^	20.16 ± 0.57 ^aA^	19.62 ± 0.21 ^aA^	19.52 ± 0.48 ^aA^
	7.5	20.46 ± 0.35 ^aA^	20.58 ± 0.64 ^aA^	20.87 ± 0.25 ^aA^	20.91 ± 0.29 ^aA^	20.11 ± 0.68 ^aA^	20.60 ± 0.43 ^aA^
180 °C Whole Wheat Flour	0	25.69 ± 0.31 ^a^	25.69 ± 0.31 ^a^	25.69 ± 0.31 ^a^	25.69 ± 0.31 ^a^	25.69 ± 0.31 ^a^	25.69 ± 0.31 ^a^
	2.5	26.04 ± 0.78 ^aA^	24.81 ± 0.10 ^aA^	25.59 ± 0.99 ^aA^	26.27 ± 0.92 ^aA^	25.84 ± 0.49 ^aA^	24.58 ± 0.57 ^bA^
	5.0	26.64 ± 0.32 ^aA^	25.72 ± 0.46 ^aAB^	25.5 ± 1.18 ^aAB^	26.52 ± 0.29 ^aA^	26.12 ± 0.14 ^aAB^	24.65 ± 0.29 ^bB^
	7.5	26.21 ± 0.56 ^aA^	25.17 ± 1.82 ^aA^	26.79 ± 0.31 ^aA^	25.84 ± 0.4 ^aA^	26.44 ± 0.31 ^aA^	25.24 ± 0.35 ^abA^
200 °C White Flour	0	20.26 ± 0.76 ^a^	20.26 ± 0.76 ^a^	20.26 ± 0.76 ^a^	20.26 ± 0.76 ^a^	20.26 ± 0.76 ^a^	20.26 ± 0.76 ^a^
	2.5	20.79 ± 1.54 ^aA^	21.31 ± 0.27 ^aA^	21.29 ± 0.82 ^aA^	20.54 ± 0.26 ^aA^	20.36 ± 0.87 ^aA^	17.05 ± 1.33 ^bB^
	5.0	20.20 ± 0.52 ^aA^	20.43 ± 0.42 ^aA^	20.30 ± 0.15 ^aA^	20.81 ± 0.32 ^aA^	20.39 ± 0.65 ^aA^	21.30 ± 1.16 ^aA^
	7.5	19.95 ± 0.15 ^aB^	21.52 ± 0.41 ^aAB^	22.68 ± 1.54 ^aA^	20.89 ± 0.2 ^aAB^	20.36 ± 0.79 ^aB^	22.83 ± 0.53 ^aA^
200 °C Whole Wheat Flour	0	26.38 ± 0.63 ^a^	26.38 ± 0.63 ^ab^	26.38 ± 0.63 ^a^	26.38 ± 0.63 ^a^	26.38 ± 0.63 ^a^	26.38 ± 0.63 ^a^
	2.5	25.54 ± 0.53 ^aA^	26.27 ± 0.58 ^abA^	25.74 ± 0.08 ^aA^	25.64 ± 0.23 ^aA^	25.76 ± 0.50 ^aA^	25.21 ± 0.54 ^abA^
	5.0	25.55 ± 0.29 ^aAB^	25.65 ± 0.42 ^bA^	26.34 ± 0.44 ^aA^	25.66 ± 1.13 ^aA^	27.01 ± 0.02 ^aA^	23.63 ± 0.60 ^bB^
	7.5	26.13 ± 0.33 ^aA^	26.97 ± 0.29 ^aA^	26.24 ± 0.68 ^aA^	25.84 ± 1.28 ^aA^	27.25 ± 0.74 ^aA^	26.06 ± 0.19 ^aA^

^1^ Means ± SD are presented. Data expressed as g/100 g on a fresh weight basis; different lowercase superscript letters in a column and different uppercase superscript in a row denote significant differences, *p* < 0.05.

**Table 5:** Lightness of date powder and cookie ^1^ samples.

		White Flour		Whole Wheat Flour			
Lightness		93.27 ^A^		87.00 ^B^			
		*Khalas*	*Khinaizi*	*Sukkari*	*Shaham*	*Zahidi*	*Fardh*
		37.72 ^F^	42.78 ^B^	45.13 ^A^	42.40 ^C^	37.90 ^E^	41.99 ^D^
Baking temp flour type and addition level							
180 °C White Flour	0	70.20 ± 2.28 ^a^	70.20 ± 2.28 ^a^	70.20 ± 2.28 ^a^	70.20 ± 2.28 ^a^	70.20± 2.28 ^a^	70.20 ± 2.28 ^a^
	2.5	48.32 ± 0.76 ^bD^	50.27 ± 1.27 ^bBC^	50.83 ± 0.10 ^bB^	48.28 ± 0.72 ^bD^	53.13 ± 0.09 ^bA^	48.81 ± 0.33 ^bCD^
	5.0	42.54 ± 1.55 ^cBC^	41.78 ± 0.39 ^cC^	44.71 ± 0.69 ^cAB^	41.79 ± 0.84 ^cC^	45.77 ± 0.10 ^cA^	41.78 ± 1.37 ^cC^
	7.5	40.55 ± 1.56 ^cA^	39.39 ± 0.33 ^cA^	41.39 ± 0.06 ^cA^	38.73 ± 0.47 ^cA^	39.61 ± 1.24 ^dA^	38.70 ± 0.52 ^cA^
180 °C Whole Wheat Flour	0	60.25 ± 0.25 ^a^	60.25 ± 0.25 ^a^	60.25 ± 0.25 ^a^	60.25 ± 0.25 ^a^	60.25 ± 0.25 ^a^	60.25 ± 0.25 ^a^
	2.5	49.70 ± 0.55 ^bA^	47.21 ± 0.38 ^bB^	47.71 ± 0.79 ^bB^	47.20 ± 1.01 ^bB^	47.60 ± 0.31 ^bB^	47.52 ± 0.41 ^bB^
	5.0	42.36 ± 1.31 ^cAB^	41.99 ± 0.32 ^cAB^	43.32 ± 0.56 ^cA^	41.26 ± 0.28 ^cB^	42.99 ± 0.30 ^cA^	42.52 ± 0.10 ^bAB^
	7.5	40.06 ± 0.70 ^dAB^	38.05 ± 0.43 ^dAB^	38.62 ± 0.52 ^dAB^	38.81 ± 0.36 ^dAB^	41.79 ± 1.07 ^cA^	35.03 ± 4.30 ^cB^
200 °C White Flour	0	71.14 ± 1.40 ^a^	71.14 ± 1.40 ^a^	71.14 ± 1.40 ^a^	71.14 ± 1.40 ^a^	71.14 ± 1.40 ^a^	71.14 ± 1.40 ^a^
	2.5	46.44 ± 0.33 ^bD^	50.02 ± 0.56 ^bB^	48.86 ± 0.60 ^bBC^	48.60 ± 0.58 ^bC^	52.11 ± 0.16 ^bA^	47.91 ± 0.52 ^bC^
	5.0	40.66 ± 1.32 ^cB^	42.38 ± 0.44 ^cB^	45.02 ± 0.40 ^cA^	42.22 ± 0.12 ^cB^	46.28 ± 0.60 ^cA^	41.48 ± 0.20 ^cB^
	7.5	39.96 ± 1.14 ^cAB^	39.70 ± 0.44 ^dABC^	40.29 ± 0.48 ^dA^	37.98 ± 0.84 ^dC^	38.37 ± 0.49 ^dABC^	38.29 ± 0.52 ^dBC^
200 °C Whole Wheat Flour	0	58.10 ± 1.79 ^a^	58.10 ± 1.79 ^a^	58.10 ± 1.79 ^a^	58.10 ± 1.79 ^a^	58.10 ± 1.79 ^a^	58.10 ± 1.79 ^a^
	2.5	47.89 ± 1.04 ^bA^	47.59 ± 0.27 ^bA^	46.44 ± 0.32 ^bA^	47.02 ± 1.11 ^bA^	47.19 ± 1.36 ^bA^	46.14 ± 0.97 ^bA^
	5.0	41.30 ± 0.25 ^cB^	42.10 ± 0.30 ^cB^	43.25 ± 0.04 ^cA^	42.02 ± 0.52 ^cB^	43.46 ± 0.65 ^cA^	41.32 ± 0.44 ^cB^
	7.5	38.07 ± 2.71 ^cA^	38.25 ± 0.71 ^dA^	37.27 ± 0.62 ^dA^	38.96 ± 0.21 ^dA^	39.48 ± 0.40 ^dA^	37.05 ± 0.37 ^dA^

^1^ Means ± SD are presented. Different lowercase superscript letters in a column and different uppercase superscript in a row denote significant differences, *p* < 0.05.

**Table 6:** Hardness assessments of cookies (N).

Baking Temp Flour Type and Addition Level		*Khalas*	*Khinaizi*	*Sukkari*	*Shaham*	*Zahidi*	*Fardh*
180 °C White Flour	0	22.82 ± 1.05 ^a^	22.82 ± 1.05 ^a^	22.82 ± 1.05 ^a^	22.82 ± 1.05 ^a^	22.82 ± 1.05 ^a^	22.82 ± 1.05 ^a^
	2.5	19.94 ± 2.02 ^abABC^	18.40 ± 0.16 ^bBC^	16.73 ± 1.33 ^bC^	22.10 ± 0.59 ^aAB^	23.02 ± 2.01 ^aA^	19.90 ± 1.20 ^bABC^
	5.0	17.72 ± 0.19 ^bA^	17.41 ± 1.78 ^bA^	16.28 ± 1.57 ^bA^	19.50 ± 2.56 ^aA^	17.59 ± 2.43 ^aA^	18.82 ± 0.68 ^bA^
	7.5	17.81 ± 0.22 ^bAB^	18.02 ± 0.97 ^bAB^	14.71 ± 0.14 ^bB^	19.31 ± 0.49 ^aA^	18.33 ± 3.12 ^aAB^	15.11 ± 0.85 ^cB^
180 °C Whole Wheat Flour	0	11.04 ± 1.20 ^a^	11.04 ± 1.20 ^a^	11.04 ± 1.20 ^a^	11.04 ± 1.20 ^a^	11.04 ± 1.20 ^a^	11.04 ± 1.20 ^a^
	2.5	10.05 ± 0.89 ^aA^	10.62 ± 0.62 ^aA^	10.81 ± 0.60 ^abA^	10.83 ± 0.72 ^aA^	9.36 ± 1.38 ^aA^	9.43 ± 0.46 ^bA^
	5.0	9.70 ± 0.36 ^aA^	10.40 ± 0.35 ^aA^	9.21 ± 0.45 ^abA^	9.90 ± 0.41 ^aA^	9.60 ± 1.67 ^aA^	9.23 ± 0.38 ^bA^
	7.5	9.06 ± 0.39 ^aA^	9.74 ± 0.49 ^aA^	8.53 ± 1.11 ^bA^	9.69 ± 0.31 ^aA^	8.91 ± 1.61 ^aA^	8.14 ± 0.50 ^cA^
200 °C White Flour	0	21.90 ± 1.54 ^a^	21.90 ± 1.54 ^a^	21.90 ± 1.54 ^a^	21.90 ± 1.54 ^a^	21.90 ± 1.54 ^a^	21.90 ± 1.54 ^a^
	2.5	19.83 ± 1.76 ^abAB^	17.22 ± 0.44 ^bB^	18.02 ± 1.63 ^bB^	23.61 ± 3.07 ^aA^	21.44 ± 1.97 ^aAB^	19.55 ± 0.85 ^bAB^
	5.0	17.34 ± 0.27 ^bAB^	17.40 ± 1.29 ^bAB^	15.91 ± 0.63 ^bcB^	20.02 ± 0.45 ^aA^	17.20 ± 2.79 ^aAB^	16.33 ± 0.87 ^bB^
	7.5	18.21 ± 0.90 ^bAB^	16.05 ± 0.89 ^bBC^	13.73 ± 0.72 ^cC^	19.70 ± 1.14 ^aA^	17.05 ± 1.47 ^bAB^	13.88 ± 1.01 ^bC^
200 °C Whole Wheat Flour	0	10.94 ± 0.79 ^a^	10.94 ± 0.79 ^a^	10.94 ± 0.79 ^a^	10.94 ± 0.79 ^a^	10.94 ± 0.79 ^a^	10.94 ± 0.79 ^a^
	2.5	10.86 ± 0.28 ^abA^	11.07 ± 0.70 ^aA^	10.22 ± 0.61 ^abA^	10.74 ± 0.63 ^aA^	9.62 ± 1.30 ^aA^	9.85 ± 0.90 ^aA^
	5.0	9.42 ± 0.37 ^bcA^	10.11 ± 0.73 ^aA^	7.97 ± 0.37 ^bcA^	9.94 ± 0.54 ^aA^	9.85 ± 1.38 ^aA^	9.06 ± 0.35 ^aA^
	7.5	9.03 ± 0.68 ^cAB^	9.34 ± 0.55 ^aA^	8.31 ± 0.91 ^cAB^	9.62 ± 0.64 ^aA^	9.33 ± 1.43 ^aA^	6.84 ± 1.06 ^bB^

Different lowercase superscript letters in a column and different uppercase superscript in a row denote significant differences, *p* < 0.05.

## Data Availability

Not applicable.
